# Reliability and validity of the Chinese version of the patient self-advocacy scale among colorectal cancer patients

**DOI:** 10.3389/fpsyg.2026.1771287

**Published:** 2026-05-08

**Authors:** Huimin Du, Dun Liu, Dandan Lü, Mengmeng Fan, Yanting Lou, Xinchu Luo

**Affiliations:** School of Nursing, Fujian Medical University, Fuzhou, China

**Keywords:** care needs, colorectal cancer, cross-cultural validation, reliability, self-advocacy, validity

## Abstract

**Background:**

Patient self-advocacy is crucial with respect to patients’ ability to participate in health care decision-making and express their individual needs and preferences, thus facilitating the establishment of better doctor–patient relationships. However, effective and concise assessment tools that can be used among cancer patients in China are lacking.

**Objectives:**

This study aims to adapt and validate a Chinese version of the Patient Self-Advocacy Scale (PSAS) for use among colorectal cancer patients.

**Method:**

The Chinese version of the PSAS was translated on the basis of the Brislin translation model. From March to August 2024, 364 colorectal cancer patients were selected from a tertiary cancer hospital through random sampling. Item analysis was conducted by reference to the critical ratio (CR) and item-total correlation; reliability was evaluated in terms of Cronbach’s alpha coefficient and the test–retest method; and an exploratory factor analysis (EFA) and a confirmatory factor analysis (CFA) were conducted to test the construct validity of the scale.

**Results:**

The overall Cronbach’s alpha coefficient for the Chinese version of the PSAS was 0.817 (0.703–0.876 for each dimension), and an excellent test–retest intraclass correlation coefficient (ICC) of 0.996 was obtained (*p* < 0.001). All the items exhibited good discriminability (critical ratio: 6.761–18.836; *p* < 0.001) and item-total correlation (0.432–0.694; p < 0.001). An exploratory factor analysis (EFA) revealed a three-factor structure, which was confirmed by a confirmatory factor analysis (CFA), thus indicating an acceptable model fit (*χ*^2^/df = 2.967, IFI = 0.927, GFI = 0.903, CFI = 0.926, RMSEA = 0.090). The scale also exhibited satisfactory content validity (I-CVI = 0.727–1.00; S-CVI = 0.939).

**Discussion:**

The adapted Chinese version of the PSAS exhibits good psychometric properties, thus making it a suitable tool for assessing the self-advocacy ability of colorectal cancer patients. This tool can serve as an effective assessment method that can support efforts to obtain a clinical understanding patients’ levels of self-advocacy and optimize doctor–patient communication approaches.

## 1 Introduction

Self-advocacy, which refers to the ability of individuals with cancer to overcome challenges in the process of realizing their preferences, needs, and values, is a crucial aspect of self-determination (Thomas et al., 2021). Such advocacy is characterized by an active process of internalizing skills and resources, which empowers individuals to meet their needs and achieve their goals. As cancer treatment evolves toward a chronic care model that involves greater patient involvement, disease management strategies have undergone significant changes (Hagan and Donovan, 2013). Self-advocacy, as an important protective factor in this context, can significantly improve patients’ quality of life and well-being by eliciting a sense of control and encouraging active participation (Egmose et al., 2023). Research has indicated that patients who possess strong self-advocacy skills are more inclined to seek essential support and tailor their medical treatments to their specific needs, thus leading to more positive health outcomes and prognoses (Hagan et al., 2018). Furthermore, self-advocacy motivates patients to actively seek information, engage with health care providers as partners, and make use of community support networks. This proactive approach enhances adherence to treatment plans, improves symptom management, and increases overall patient satisfaction, thereby potentially reducing the burden on health care resources and enabling patients to play a more active role in their health journey, ultimately resulting in more personalized and effective care (Zhang et al., 2022; Hagan and Donovan, 2013).

Self-advocacy is particularly crucial for colorectal cancer patients, who face unique challenges in terms of health knowledge (Dau et al., 2020; Xiangting et al., 2023), medical decision-making (Gu et al., 2023), and psychological support (Cheng et al., 2022). The impact of this disease on patients’ intestinal functions, including digestion, absorption, and excretion, especially in cases involving stomas, necessitates specialized information to support effective daily life management and informed medical decision-making (Wyer, 2022). In the context of medical decision-making, the self-advocacy exhibited by colorectal cancer patients has certain distinct characteristics. For example, such patients may advocate surgical procedures that preserve anal function to increase their quality of life postoperation, as surgery can significantly impact intestinal function (Piozzi et al., 2021).

Additionally, treatments can alter patients’ intestinal flora, thus necessitating discussions with health care providers to mitigate potential symptoms such as diarrhea and bloating (Wong and Yu, 2023). Furthermore, in terms of social support and psychological adjustment, these patients often face stress and discrimination because of changes in their body image, such as the presence of a stoma. It is essential for them to raise social awareness regarding accessible stoma care facilities and to seek counseling to help address any distress related to body image (Li G. et al., 2024; Li L. et al., 2024; Li S. et al., 2024).

The Patient Self-Advocacy Scale (PSAS), which was developed by Brashers et al. (1999), is a psychometric instrument designed to measure patient engagement in health care decision-making, which serves as a key aspect of self-advocacy within the patient empowerment framework (Brashers et al., 1999). For colorectal cancer patients, who face complex medical decisions and require specialized health knowledge, the PSAS serves as a structured tool that can be used to evaluate their ability to manage these challenges. By identifying areas of low engagement or self-advocacy, the PSAS can help health care providers tailor support enhance patient autonomy and decision-making. This targeted support empowers patients to play a more proactive role in their care and fosters collaborative relationships with health care providers, thus leading to improved treatment adherence, symptom management, and patient satisfaction. The cultural adaptability of the PSAS, which has been demonstrated by its successful translation and application in diverse populations, such as those with chronic heart failure (Kleman and Ross, 2023) and AIDS (Brashers et al., 1999), validates its broad applicability across various health conditions and cultural settings. Unlike scales such as the Female Self-Advocacy in Cancer Survivorship Scale (FSACS), which focuses exclusively on female perspectives (Deng et al., 2022), the PSAS includes both male and female perspectives, thus increasing its relevance for investigations of self-advocacy across different cancer groups, including those with colorectal, lung, or prostate cancer. Considering the underutilization of international self-advocacy scales among cancer patients and the scarcity of culturally adapted scales in China, this study aims to adapt the PSAS for exclusive use among colorectal cancer patients, thereby addressing a significant gap in self-advocacy assessment for this cohort.

## 2 Materials and methods

The present study aimed to establish the equivalence between the original Chinese and English versions of the PSAS in terms of operability, semantics, concept, and measurability across two stages. The first stage involved generating a Chinese version of the PSAS through a process involving translation and cross-cultural modifications, followed by a cross-sectional survey in the second stage to verify the reliability and validity of this tool. This study was conducted in accordance with the principles of the Declaration of Helsinki and was approved by the Biomedical Research Ethics Review Committee of Fujian Medical University (Approval No.: “2024,” 82).

### 2.1 Translation and cross-cultural adaptation

This study employed the Brislin translation model to adapt the scale into Chinese after authorization was provided by the original author (He et al., 2023). The process included the following steps: (1) Direct translation. The initial translation was conducted by a master’s student and a doctoral student in nursing, both of whom were native Chinese speakers who were also proficient in English; this step resulted in two Chinese versions of the measure. (2) Synthesis of translations. The research team compared the two preliminary Chinese versions, and any unresolved discrepancies were addressed through consultation with the original authors; this step led to the production of an initial Chinese version. (3) Back-translation. Two English master’s students, who were unfamiliar with the original PSAS scale, independently translated the initial Chinese version back into English, resulting in two English versions, which were labeled PSAS-B1 and PSAS-B2. The team meticulously aligned these translations with the original version, iteratively refining them to ensure semantic equivalence. The inaugural Chinese version of the self-advocacy scale was established following a review and validation by the original authors. (4) Cross-cultural adaptation. The inaugural Chinese version of the Patient Self-Advocacy Scale included a cultural adaptation process that was facilitated by a process of expert consultation that involved three academic nursing faculty members, two practicing clinicians, and six nursing administrators. Each expert commented on the semantics, expression, content, and cultural background of the items included in the questionnaire on the basis of a 4-point Likert scoring system, in which context a score of 1 indicated “irrelevant,” 2 signified a “weak correlation,” 3 denoted a “strong correlation,” and 4 represented a “very strong correlation”. (5) Presurvey. Convenience sampling was used to recruit 30 colorectal cancer patients to participate a preliminary evaluation of the semantic and lexical accuracy of the Chinese version of this instrument. Following this assessment, terms that were unclear or ambiguous were refined. The optimized Patient Self-Advocacy Scale was then implemented in the formal survey.

### 2.2 Reliability and validity tests of the PSAS

#### 2.2.1 Study design and participants

A cross-sectional study was conducted on the basis of a random sampling approach. Patients who were diagnosed with colorectal cancer (CRC) were recruited from a tertiary care cancer hospital between March and August 2024. The inclusion criteria were as follows: (1) patients aged 18 years or older; (2) patients with good verbal communication skills and no impairments of cognitive function; and (3) patients who understood their medical condition and voluntarily participated in the survey.

In accordance with the principles used to determine the appropriate sample size for item analyses and exploratory factor analyses (Jian et al., 2024), the sample size should be 5 to 10 times the number of scale items. For confirmatory factor analyses, the sample size should be no less than 200 cases. Additionally, considering a 10% attrition rate, a minimum of 286 cases was needed. Ultimately, a total of 364 CRC patients who met the inclusion criteria were reexamined.

#### 2.2.2 Instruments

##### 2.2.2.1 Demographic characteristics

The research tools used in this study included a general questionnaire and the Chinese version of the Patient Self-Advocacy Scale (PSAS). The general questionnaire included two sections: basic information, such as sex, age, marital status, education level, current employment status, monthly income, place of residence, and medical expenses, and clinical information, including tumor type, tumor location, current tumor stage, treatment options, ostomy, oral chemotherapy, and the presence of any other chronic conditions.

##### 2.2.2.2 The Chinese version of the PSAS

The original Patient Self-Advocacy Scale (PSAS), which was developed by Brashers et al. (1999) to measure patient participation in the context of health care decision-making interactions (Deng et al., 2022), initially focused on the male population with AIDS. It includes three dimensions, i.e., increased disease education (four items), increased assertiveness (four items), and the possibility of noncompliance (four items), for a total of 12 items. Each dimension can be applied independently. These items are scored on a 7-level point Likert scale, in which 1 signifies “strongly disagree” and 7 signifies “strongly agree.” Higher scores indicate greater patient self-advocacy. The Cronbach’s alpha coefficient for this scale is 0.78, thus indicating good internal consistency. In our study, we used the Chinese version of the patient self-advocacy scale, which was revised following expert consultation and a pilot survey.

### 2.3 Data collection

Eligible patients were enrolled after the surveyors explained the purpose of the study to these individuals and obtained their consent. Participants were subsequently given a structured questionnaire that contained both general information questions and the PSAS. Investigators checked the questionnaires on the spot for missing entries or logical errors to ensure the accuracy and completeness of the data.

### 2.4 Data analysis

SPSS Statistics 26 and Amos 28 graphics software were used to support the statistical analysis. In accordance with the study’s objectives, a variety of statistical methods, including descriptive statistical analysis, item analysis, and reliability and validity analysis, were employed to process the data.

#### 2.4.1 Descriptive statistical analysis

The general data of the patients were analyzed on the basis of descriptive statistical methods. Specifically, age is described as the mean ± standard deviation, whereas other variables, such as sex, tumor type, and tumor site, are described in terms of frequencies and percentages.

#### 2.4.2 Item validity

##### 2.4.2.1 Critical ratio method

The total scores of the 364 patients on the Chinese version of the PSAS scale were ranked in ascending order. The lowest 27% of the scores were assigned to the low group, and the highest 27% were assigned to the high group. Independent sample t tests were performed. A higher critical ratio (CR) was taken to indicate a greater degree of item discrimination. If the CR > 3 and *p* < 0.05, the item in question was identified as highly discriminative. If *p* > 0.05, the corresponding item was deleted (Wang et al., 2024).

##### 2.4.2.2 Correlation coefficient method

Pearson correlation analysis was conducted to evaluate the correlation coefficients between each item and the total score on the Chinese PSAS. A correlation coefficient (*r*) greater than 0.4 and a *p*-value less than 0.05 were taken to indicate good item correlations; conversely, if *r* was less than 0.4 or if the *p*-value exceeded 0.05, the corresponding item was removed (Wang et al., 2024).

#### 2.4.3 Reliability analysis

##### 2.4.3.1 Internal consistency reliability

Cronbach’s alpha coefficient was used to evaluate the internal consistency of the scale. If the Cronbach’s alpha coefficient exceeded 0.7, the scale’s reliability was determined to be good (Zhang et al., 2024).

##### 2.4.3.2 Test–retest reliability

After a two-week interval, 38 patients were once again asked to complete the same questionnaire; these patients were selected through purposive sampling. The intraclass correlation coefficient (ICC) was used to represent the retest reliability of the scale. If the ICC exceeded 0.75, such a finding would indicate that the scale’s retest reliability was excellent (Wang et al., 2024; Zhang et al., 2024).

#### 2.4.4 Validity analysis

##### 2.4.4.1 Content validity

The evaluation of content validity focused on the individual item content validity index (I-CVI) and the scale’s average content validity index (S-CVI). If the I-CVI was greater than or equal to 0.78 and the S-CVI was greater than or equal to 0.9, then the content validity was determined to be satisfactory (Chen et al., 2024).

##### 2.4.4.2 Structural validity

An exploratory factor analysis (EFA) and a confirmatory factor analysis (CFA) were conducted to assess the structural validity of the Chinese version of the PSAS. To ensure the rationality and robustness of the process of factor structure exploration and verification, the total sample of 364 participants was randomly divided into two groups for EFA and CFA at a ratio of approximately 1:2 (i.e., 120 cases for the EFA and 244 cases for the CFA). This ratio is in line with the sample size criteria recommended in psychometric research: EFA requires a sample size of 5–10 times the number of scale items, and CFA requires no fewer than 200 cases (Jian et al., 2024; Li G. et al., 2024; Li L. et al., 2024; Li S. et al., 2024). This ratio is also commonly used in scale validation studies and can establish a good balance between the sample size requirements of the two analytical stages.

In the EFA, factor analysis was considered to be suitable when the Kaiser–Meyer–Olkin (KMO) measure of sampling adequacy was greater than 0.8 and the result of Bartlett’s test of sphericity was statistically significant (Li G. et al., 2024; Li L. et al., 2024; Li S. et al., 2024). Principal component analysis (PCA) was selected as the extraction method for EFA instead of common factor analysis, given that PCA is more suitable for the initial exploration of scale factor structure due to its advantages of simple calculation, clear factor interpretation and high extraction efficiency with regard to common variance (García-Dopico et al., 2023; Van Horn et al., 2023). EFA was conducted on the basis of principal component analysis with varimax orthogonal rotation, and common factors that featured eigenvalues greater than 1 were extracted (García-Dopico et al., 2023). Items that exhibited factor loading values below 0.4 or cross-loading were excluded from the scale (Van Horn et al., 2023).

As part of the CFA, the maximum likelihood method was used to evaluate the model fit. A good model fit was defined as follows: a chi-square/degree of freedom (*χ*^2^/df) ratio < 3.000, a root mean square error of approximation (RMSEA) < 0.080, and a goodness-of-fit index (GFI), a comparative fit index (CFI), and an incremental fit index (IFI) > 0.900 (Al-Habbal et al., 2022).

## 3 Results

### 3.1 Translation and cross-cultural adaptation

During the two rounds of expert consultation, the experts exhibited a 100% positive response rate. With respect to the expert consultation pertaining to specific items, regarding Item 1, “I believe that it is important for people to learn as much as they can about their illness and treatments,” the experts exhibited disagreement. Namely, some experts believed that the term “they” should not be changed to “I,” whereas others held the opposite view. Following consultation with the original author, the term “they” was changed to “I”; thus, Item 1 was ultimately revised to “I believe that it is important for me to learn as much as I can about my illness and treatments.” With respect to Items 6 and 7, the experts also exhibited disagreement. Some experts considered the term “health care needs” to be synonymous with “health care,” but others believed that a distinction should be made between these concepts. Following consultation with the original author and efforts to understand the difference, the meaning of “health care needs” was retained.

### 3.2 Pre-investigations

During the pre-investigation phase, 30 questionnaires were distributed, and all of those questionnaires were collected, for a valid response rate of 100%. The time required to complete the questionnaire ranged from 4 to 6 min. All the respondents agreed that the semantics of the items included in the questionnaire were clear and easy to understand; hence, the content remained unchanged after the presurvey.

### 3.3 Descriptive statistics

A total of 364 colorectal cancer patients were included in this study. The majority of these participants were male (68.13%), unemployed (56.32%), married (93.13%), and had an education level below middle school (69.23%). The mean age of the participants was 55.53 ± 10.65 years. Additionally, adenocarcinoma was present in approximately 97.25% of patients, with 54.40% of these individuals presenting with rectal cancer and 62.74% of cases being classified as intermediate to advanced. The detailed characteristics of these CRC patients are detailed in Table 1.

**Table 1:** Characteristics of the participants (*N* = 364).

Characteristics	*N*	%
Gender		
Male	248	68.13
Female	116	31.87
Age (mean, standard deviation)	55.53 ± 10.65	
Marital status		
Married	339	93.13
Unmarried	6	1.65
Divorced/widowed	19	5.22
Education level		
Junior high school or below	252	69.23
High school	55	15.11
College or above	57	15.66
Current employment status		
Employed	118	32.42
Unemployed	205	56.32
Retired	41	11.26
Average monthly family income		
Below the regional average	169	46.43
Above the regional average	195	53.57
Place of residence		
Urban	179	49.18
Rural	185	50.82
Medical expenses		
Medical insurance	360	98.81
Self-financing	4	1.19
Tumor type		
Adenocarcinoma	354	97.25
Adenosquamous carcinoma	10	2.75
Tumor location		
Rectum	198	54.40
Sigmoid colon	92	25.27
Ascending colon	38	10.44
Descending colon	19	5.22
Transverse colon	12	3.3
Other	5	1.37
Neoplasm staging		
I	51	14.01
II	29	7.97
III	112	30.67
IV	116	32.07
Unstaged	56	15.37
Treatment plan		
Surgery	93	25.55
Chemotherapy	113	31.05
Surgery + Chemotherapy	92	25.27
Surgery + Targeted therapy + Chemotherapy	22	6.04
Targeted therapy + Chemotherapy	35	9.62
Surgery + Targeted therapy	9	2.47
Stoma		
Yes	37	10.16
No	327	89.84
Oral chemotherapy		
Yes	104	28.57
No	260	71.43
Chronic disease		
Yes	78	21.43
No	286	78.57

### 3.4 Item analysis results

According to the critical ratio method analysis, the CR values for all the items in the low and high groups varied between 6.761 and 18.836, thus indicating that each item surpassed the recommended threshold of 3 (*p* < 0.01). Additionally, the correlation coefficient method analysis revealed that the r values of each item with respect to the total score of the scale ranged from 0.432 to 0.694 (*p* < 0.01). All the *r* values thus exceeded the recommended level of 0.4, and *p* < 0.01 (Chen et al., 2024). Consequently, all 12 items were deemed suitable for further analysis. Table 2 presents the comparison of scores between the high- and low-score groups, and Table 3 provides a comprehensive summary of the Pearson correlations between items and total scores.

**Table 2:** Comparison of scores between the high-score and low-score groups (*N* = 364).

Item	Low-score group (*n* = 100), mean (SD)	High-score group (*n* = 105), mean (SD)	*t*-test (df)	*p-*value
1	4.66(1.43)	6.90(0.405)	15.131(114.060)	<0.001
2	3.63(1.495)	6.41(1.190)	14.680(189.038)	<0.001
3	3.02(1.333)	6.11(1.003)	18.836(203)	<0.001
4	2.06(1.293)	4.64(1.442)	13.452(203)	<0.001
5	4.26(1.346)	6.59(0.917)	14.423(173.579)	<0.001
6	3.15(1.431)	5.90(1.297)	14.452(203)	<0.001
7	3.54(1.487)	5.10(1.724)	6.902(203)	<0.001
8	3.25(1.604)	5.17(1.863)	7.896(203)	<0.001
9	5.11(2.079)	6.66(1.036)	6.693(143.735)	<0.001
10	5.15(2.167)	6.72(0.872)	6.761(128.946)	<0.001
11	4.97(1.962)	6.61(1.236)	7.119(165.537)	<0.001
12	5.29(2.133)	6.81(0.722)	6.763(120.403)	<0.001

**Table 3:** Pearson correlations between the items and total scores (*N* = 364).

Item	Pearson correlation	*p-*value
1	0.672	<0.001
2	0.641	<0.001
3	0.694	<0.001
4	0.587	<0.001
5	0.674	<0.001
6	0.676	<0.001
7	0.432	<0.001
8	0.494	<0.001
9	0.513	<0.001
10	0.531	<0.001
11	0.499	<0.001
12	0.569	<0.001

### 3.5 Results of the reliability test of the Chinese patient self-advocacy scale

The Chinese version of the PSAS exhibited a high degree of internal consistency, as indicated by a Cronbach’s alpha coefficient of 0.817 for the overall scale and corresponding coefficients ranging from 0.703 to 0.876 for each dimension. Table 4 presents the corrected item–total correlation and the Cronbach’s alpha coefficient if the item were to be removed. Following a two-week interval, the test–retest reliability of the scale was established, as indicated by an intraclass correlation coefficient (ICC) of 0.996 (*p* < 0.001), which exceeded the threshold of 0.7.

**Table 4:** Corrected item–total correlations and Cronbach’s alpha coefficients if the item was deleted (*N* = 364).

Item	Corrected item total correlation	Cronbach’s alpha if the item was deleted
1	0.602	0.795
2	0.539	0.797
3	0.603	0.791
4	0.576	0.803
5	0.678	0.705
6	0.696	0.794
7	0.584	0.783
8	0.698	0.808
9	0.593	0.810
10	0.610	0.809
11	0.577	0.811
12	0.665	0.804

### 3.6 Results of the validity test of the PSAS

#### 3.6.1 Content validity

A panel of 11 experts evaluated the content validity of the Chinese version of the PSAS. The findings indicated an item-level content validity index (I-CVI) that ranged from 0.727 to 1.00, alongside a scale-level content validity index (S-CVI) of 0.939, which met the satisfactory content validity criteria (I-CVI ≥ 0.70, S-CVI ≥ 0.90; Chen et al., 2024).

#### 3.6.2 Structural validity

##### 3.6.2.1 Results of the exploratory factor analysis

**Figure 1:**
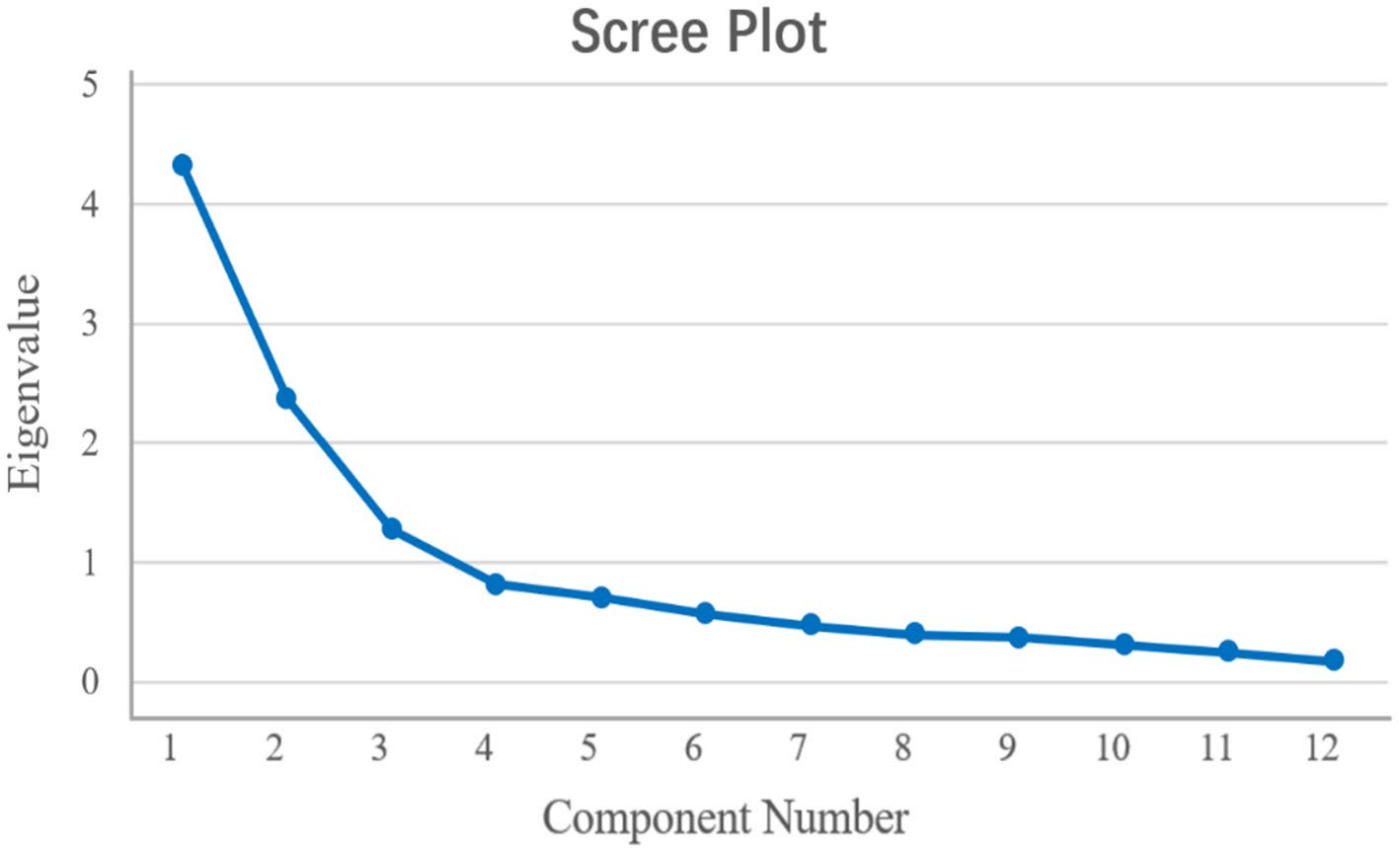
Scree plot of the exploratory factor analysis pertaining to the Chinese version of the PSAS.

The EFA was conducted by reference to a sample of 120 individuals. The Kaiser–Meyer–Olkin statistic was 0.792, and the value revealed by Bartlett test of sphericity was 636.578. Therefore, the results of the data analysis were deemed appropriate for conducting the EFA. On the basis of a principal component analysis with varimax rotation, three distinct common factors were revealed; these factors explained 66.256% of the total variance. Table 5 provides a comprehensive overview of these findings. Additionally, the scree plot presented in Figure 1 suggests a decreasing trend after the third point, thus providing further support for the three-factor structure.

**Table 5:** Exploratory factor analysis (*N* = 120).

Items	Factor 1	Factor 2	Factor 3
1. I believe that it is important for me to learn as much as I can about my illness and treatments.		0.777	
2. When I am sick, I actively seek out information regarding my illness.		0.777	
3. I am more educated about my health than most Chinese citizens are.		0.738	
4. I have full knowledge of the health problems faced by people like me.		0.783	
5. I get what I need from my physician because I am assertive.			0.738
6. I am more assertive about my health care needs than most Chinese citizens are.			0.603
7. I frequently make suggestions to my physician about my health care needs.			0.646
8. If my physician prescribes me something that I do not understand or agree with, I question it.			0.748
9. Sometimes there are good reasons not to follow the advice of a physician.	0.885		
10. Sometimes I think that I have a better grasp of my needs medically than my doctor does.	0.886		
11. If I’m given a treatment by my physician with which I do not agree, I am likely not to take it.	0.779		
12. I do not always do what my physician or health care worker has asked me to do.	0.833		

##### 3.6.2.2 Results of the confirmatory factor analysis

**Figure 2:**
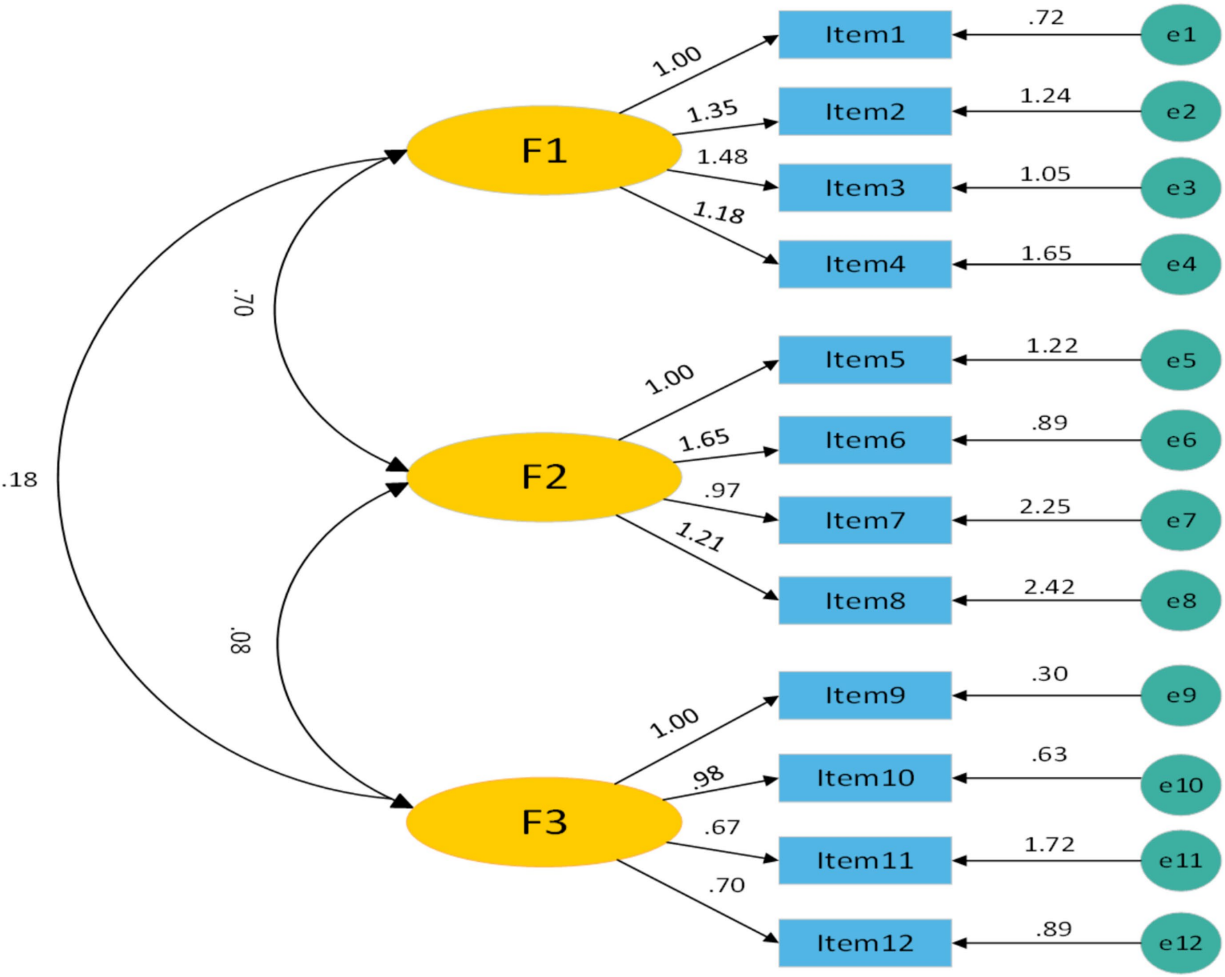
Confirmatory factor analysis (*N* = 244). F1 represents Increased Education (IE) and consists of four items; F2 represents Increased Assertiveness (IA) and also consists of four items; and F3 represents the Potential for Mindful Non-adherence (PfN) and consists of four items.

Following the EFA results, a CFA model was constructed by reference to a sample of 244 cases; this model included three distinct common factors and exhibited an acceptable fit to the data. The detailed statistical results are presented in Figure 2.

## 4 Discussion

In this study, we meticulously adapted the Patient Self-Advocacy Scale (PSAS) for use in China, in which context we used the Brislin model to ensure linguistic and cultural accuracy, as well as applicability. Our approach was systematic and multidisciplinary, and it emphasized the importance of cultural adaptation in enhancing scale utility within local contexts. After several rounds of expert consultation, we reviewed the Chinese expressions used for the terms included in the scale to ensure clarity and cultural appropriateness. This rigorous process not only ensured linguistic accuracy but also improved the scale’s applicability within the target culture, thus ensuring its scientific and practical utility among the target population.

According to classical test theory (CTT), a reliable scale must exhibit both internal consistency and test–retest reliability (Tong et al., 2024), and our findings are in line with these theoretical expectations, thus confirming the reliability of the Chinese version of the PSAS. To assess internal consistency, we calculated the Cronbach’s alpha coefficient, which was 0.817, i.e., well above the widely accepted threshold of 0.7, thus indicating good internal consistency; notably, this value is higher than that of the original scale, which was developed by reference to a sample of 174 HIV patients. In contrast, our study included colorectal cancer patients undergoing surgery and chemotherapy, who commonly experience considerable uncertainty regarding treatment outcomes and fear of cancer recurrence, and this specific clinical context may have contributed to the relatively high Cronbach’s alpha identified in this context, as these patients were more motivated to obtain health information and interact with health care providers with the goal of enhancing their disease education and confidence in self-management. With respect to the exceptionally high test–retest reliability coefficient of 0.996 observed in this context, this study strictly complied with rigorous psychometric and clinical research protocols, including by ensuring the complete cross-cultural adaptation of the PSAS through forward translation, back-translation, expert review, and pilot survey optimization, thus ensuring clear and unambiguous item semantics. In addition, standardized instructions, uniform testing environments, and trained researchers were used in both the test and retest phases to minimize measurement errors. The retest sample consisted of 38 colorectal cancer patients who were strictly selected and who exhibited intact cognitive function and a consistent understanding of the scale items; furthermore, the 2-week retest interval considered in this research represents a conventional and reasonable timeframe in clinical behavioral research that can help prevent excessive memory effects while maintaining short-term behavioral stability. Furthermore, self-advocacy is a stable long-term behavioral trait that emerges during the process by which patients adapt to chronic disease, and in the absence of targeted interventions, patients’ disease status and living environment remained stable during the 2-week period, thus leading to minimal fluctuations in their levels of self-advocacy and, accordingly, high consistency between the two measurements.

In this study, the content validity and structural validity of the Chinese version of the Patient Self-Advocacy Scale were comprehensively evaluated. Eleven experts who had extensive clinical and academic experience were invited to assess the content validity of this tool, thus ensuring that the scale revision process was rigorous and authoritative. The results revealed that the I-CVI ranged from 0.727 to 1.00 and that the S-CVI was 0.939, thus supporting the excellent content validity of the adapted scale. With respect to structural validity, an exploratory factor analysis (EFA) and a confirmatory factor analysis (CFA) were performed. The EFA extracted three common factors with eigenvalues greater than 1, which explained a total of 66.256% of the variance. According to classical factor analysis theory (Hebeshy et al., 2020), factors with eigenvalues that exceed 1 are considered core dimensions, thus indicating that the three-factor structure can effectively represent the multifaceted concept of patient self-advocacy. Although the cumulative variance explained in this context was moderate, it still provided a reasonable interpretation of scale variance and a reliable foundation for the verification of structural validity. The results of the CFA also supported an acceptable model fit. In combination with the other excellent fit indices, the overall model remained satisfactory and confirmed good structural validity; although the RMSEA value of 0.090 was slightly above the ideal cutoff of 0.08, it remained within the acceptable range, i.e., below 0.10. This slightly elevated RMSEA may be attributed to cultural differences between Western and Chinese populations. Although strict translation and cross-cultural adaptation processes were implemented, subtle discrepancies in linguistic expression, cultural values, and doctor–patient communication patterns may still have had minor influences on the factor structure. Therefore, future studies should be conducted to revise and culturally optimize scale items in further depth with the aim of enhancing model fit and structural validity.

### 4.1 Limitations

The cross-sectional design of this research precludes any assessment of the long-term trajectory of self-advocacy or its responsiveness to interventions. Generalizability may be limited by the single-center setting of this research and its focus on colorectal cancer patients, which may not fully capture cultural and regional variations. Although our study population exhibited good representativeness, including diverse urban–rural and educational backgrounds, future studies on the basis of broader samples are needed to verify the reliability and applicability of our findings (Hutchens et al., 2023). Confirmatory factor analysis indicated an acceptable model fit, although the RMSEA value of 0.090 slightly exceeded the 0.08 threshold, which may reflect the impacts of cultural nuances despite cross-cultural adaptation. Future multicenter longitudinal studies involving heterogeneous samples are warranted to validate the scale’s psychometric properties and clinical utility in further detail.

## 5 Conclusion

Patient self-advocacy, a complex and multidimensional concept that is influenced by various factors, lacks specific assessment tools that can be used for all patients in China. Therefore, research on patient self-advocacy aims to measure the various levels of such advocacy. In this study, during the process of cross-cultural adaptation, rigorous adherence to the Brislin model and the Chinese Patient Self-Advocacy Scale (PSAS) was guaranteed, thereby ensuring linguistic equivalence between the original scale and the Chinese version. As an objective assessment tool for patients, the Chinese version of the PSAS can help medical staff understand the self-advocacy status of patients, thereby allowing the former to select appropriate communication methods and enhancing doctor–patient communication. Moreover, this tool can help patients improve their self-efficacy and treatment compliance through self-advocacy.

## Data Availability

The original contributions presented in the study are included in the article/supplementary material, further inquiries can be directed to the corresponding author.
